# Gene variants and expression changes of SIRT1 and SIRT6 in peripheral blood are associated with Parkinson’s disease

**DOI:** 10.1038/s41598-021-90059-z

**Published:** 2021-05-21

**Authors:** Rita Maszlag-Török, Fanni A. Boros, László Vécsei, Péter Klivényi

**Affiliations:** 1 Department of Neurology, Faculty of Medicine, Albert Szent-Györgyi Clinical Center, University of Szeged, P.O. Box: 427, 670l Szeged, Hungary; 2 MTA - SZTE Neuroscience Research Group, Szeged, Hungary

**Keywords:** Parkinson's disease, Parkinson's disease, Molecular neuroscience

## Abstract

Parkinson’s disease (PD) is a neurodegenerative disease caused by complex interaction between genetic and environmental factors. There is a growing body of evidence of the involvement of sirtuins (SIRTs) in disease pathomechanism. SIRTs are NAD+-dependent histone deacetylases which take part in various cellular functions. However, available data of the relationship between SIRT gene polymorphisms and PD is limited. Our aim was to investigate the possible association of 10 SNPs identified within non-mitochondrial SIRTs, *SIRT1, -2* and *-6* with the risk of PD in Hungarian population, and to compare the expression level of these SIRTs between healthy controls and PD patients. Our results showed that rs3740051 and rs3818292 of *SIRT1* and rs350843, rs350844, rs107251, rs350845 and rs350846 of *SIRT6* show weak association with PD risk. On the contrary rs12778366 and rs3758391 of *SIRT1* and rs10410544 of *SIRT2* did not show association with PD. Moreover, we detected that mRNA level of *SIRT1* was down-regulated, and mRNA level of *SIRT6* was up-regulated, while *SIRT2* mRNA level was not altered in the peripheral blood of PD patients as compared to controls. The difference in both cases was more pronounced when comparing the early-onset PD group to the control cohort. Nevertheless, mRNA level changes did not show any association with the presence of any of the investigated SNPs either in the PD or in the control group. In conclusion, our findings suggest that non-mitochondrial sirtuins, SIRT1 and -6 but not SIRT2 might contribute to the pathogenesis of PD in the Hungarian population both via their altered mRNA levels and via gene alterations identified as specific SNPs.

## Introduction

Parkinson’s disease (PD) is the second most common neurodegenerative disorder worldwide^1^. It is characterized by the loss of dopaminergic neurons in the *substantia nigra* pars compacta and the presence of Lewy bodies, which are accumulations of aggregated alpha-synuclein (SNCA) in the cytoplasm of surviving neurons. Although the exact pathomechanism of PD is still not fully elucidated, several molecular mechanisms leading to neuronal death thus culminating in the development of the disease have been described. These include mitochondrial dysfunction, oxidative stress, microglia activation and neuroinflammation. Regarding aetiological background, the most accepted concept is that PD results from complex interactions among molecular changes related to aging, environmental factors and genetic constitution^2^.

Sirtuins (SIRTs) are NAD+-dependent class III histone deacetylase enzymes which take part in various biological processes. There are seven SIRT homologs (SIRT1-7) showing different enzymatic specificity which are localized differently in mammalian cells. SIRT1, -6, and -7 reside primarily in the nucleus, SIRT2 is cytoplasmic, while SIRT3, -4 and -5 are predominantly localized in mitochondria. The pathways in which SIRTs are involved, such as stress response, mitochondrial dysfunction, oxidative stress, protein aggregation and inflammation, are closely linked to cell survival and are intertwined with age-related neurodegenerative diseases^3^. Mitochondrial dysfunction in particular has been strongly implicated in the pathogenesis of the neurodegenerative disorders^4^. Thus, it is not surprising that SIRTs, which affect mitochondrial function via the regulation of various metabolic processes, also affect diseases related to neurodegeneration^5^. On the other hand a growing body of evidence implicates non-mitochondrial SIRTs—such as SIRT1, -2 and -6—as well in the development and course of neurodegenerative diseases, including PD^6^. Data obtained on the effect of these SIRTs on PD in models indicate diverse roles of these enzymes in disease development, however, results are sometimes contradictory (see below).

SIRT1 expression was found to be decreased in both toxin induced and genetically modified models of PD^7^. Resveratrol, a SIRT1 activating compound was found to be neuroprotective in both 6-OHDA and MPTP models of PD, as treatment with the compound decreased dopaminergic neuron death^8–10^. Moreover, SIRT1 overexpression was found to inhibit the formation of alpha-synuclein aggregates via the activation of peroxisome proliferator-activated receptor gamma coactivator 1-alpha (PGC-1α) and molecular chaperons in neuroblastoma cells and in a mice model of the disease, respectively^11^.

Data on the role of SIRT2 in PD are contradictory: SIRT2 has been reported to exacerbate alpha-synuclein toxicity via deacetylation of alpha-synuclein^12^. On the other hand, SIRT2 knockout mice presented reduced MPTP-induced dopaminergic cell damage and inhibition of SIRT2 activity reduced alpha-synuclein toxicity thus had a protective effect on dopaminergic neurons in both in vitro and in vivo models of the disease^13,14^. However, there are also data arguing against the protective effect of SIRT2 inhibition, as in diquat and rotenone treated cells enzymatic inhibition of SIRT2 enhanced the formation of alpha-synuclein aggregates resulting in increased cell death, while elevated SIRT2 expression had a cell protective effect^15^.

Reports on the role of SIRT6 in age related diseases and neurodegeneration are similarly contradictious. Overexpression of SIRT6 was found to increase the lifespan of mice, and ameliorate certain age-associated disease (such as cancer) in rodents^16^. On the contrary, regarding its neurodegeneration modulating properties, SIRT6 resembles SIRT2. SIRT6 knockout was found to be protective in a MPTP-induced mouse model of PD, while SIRT6 overexpressing mice showed more severe disease pathology, indicated by increased dopaminergic cell death compared to wild type animals^17^.

Despite of the considerable amount of data from in vitro and in vivo models pointing to the role of SIRTs in PD, little is known about the involvement of these enzymes/genes in the disease from results obtained from studying patients in Caucasian population. Genetic variations of SIRT genes may affect the transcription regulation of the gene, or the translation and/or enzymatic activity of the protein, and various SIRT gene variants have been identified to be associated with different malignancies^18,19^. However, only limited data are available on the significance of SIRT alterations in PD, therefore studying SIRT expression and SIRT SNPs in relation to this disease is highly important.

Therefore, one of the aims of the current study was to compare the frequency of non-mitochondrial SIRT variants in Hungarian sporadic PD (SPD) patients and healthy control individuals. We selected SIRT SNPs that are intensively studied or have been identified recently as potential risk factors for PD. The frequencies of these polymorphisms vary greatly in different populations and to our knowledge their occurrence in relation to PD has not been investigated in the Central European population so far. Our study included four *SIRT1* (rs3818292, rs3758391, rs12778366, rs3740051), one *SIRT2* (rs10410544) and five *SIRT6* (rs350843, rs350844, rs350845, rs350846 and rs107251) polymorphisms. Our further goal was to determine whether changes in the expression levels of *SIRT1,-2* and *-6* genes are observable between PD patients and controls, and in relation to the presence of these SIRT gene variants.

Earlier, the frequencies of a total of 41, *SIRT1-6* genes SNPs were investigated in Spanish PD patients. No association was found between any of the polymorphisms and disease occurrence in the investigated population^20^. More recently, a case–control study reported that a SNP (rs12778366) in the promoter region of *SIRT1* and another (rs2015) in the 3′ UTR region of *SIRT2* were associated with PD in Chinese Han population. Moreover, this study reported down-regulated *SIRT1* expression, and up-regulated *SIRT2* mRNA level in PD patients as compared to non-PD controls. These gene expression changes correlated with the presence of the rs12778366 of *SIRT1* and r2015 of *SIRT2* variants^21^. Results of a meta-analysis by Nicholatos et al*.* suggested associations between the presence of six *SIRT6* SNPs and the incidence of PD. All the six polymorphisms were also associated with elevated expression level of SIRT6 in the brain tissue (from frontal or mid-temporal cortex) of PD patients in American population^17^. The findings outlined above strongly support the involvement of non-mitochondrial SIRTs (SIRT1, SIRT2 and SIRT6) in the pathogenesis of PD.

## Results

### Associations between SIRT SNPs and PD

The frequency of each polymorphism investigated in this study was in accord with Hardy–Weinberg Equilibrium (HWE) in the studied population groups. The genotype and allele frequencies of the investigated SNPs and their associations with the risk of PD are summarized in Table 1. We observed strong associations between PD and the minor allele frequencies of rs3740051 (p = 0.0212) and rs3818292 (p = 0.00978) variants of *SIRT1*, and rs350843 (p = 0.045), rs350846 (p = 0.025) of *SIRT6*. However, none of these associations were statistically significant after the FDR correction (p = 0.0832, p = 0.0832, 0.0880 and p = 0.0832 respectively). SNPs rs3740051, rs3818292, rs350843, rs350844, rs107251, rs350845 and rs350846 showed a trend of association in the dominant model, suggesting a possible link between these SNPs and the development of PD. The genotype and allele frequencies of the other investigated polymorphisms (rs12778366, rs3758391 of *SIRT1* and rs10410544 of *SIRT2*) showed no significant differences between cases and controls. Allele and genotype distributions of the *SIRT1* and *SIRT2* SNPs were also similar after stratification by age, disease onset or gender. On the other hand, 5 SNPs (rs350843, rs350844, rs107251, rs350845 and rs350846) of the *SIRT6* gene showed a significant association with EOPD (p = 0.025, p = 0.037, p = 0.037, p = 0.037, and p = 0.025). These significant differences however disappeared after FDR correction (p = 0.074, p = 0.074, 0.074, p = 0.074 and p = 0.074 respectively).

**Table 1:** Statistical results of the investigated SNP.

Gene	SNP Alleles	Genotype p-value	Genotype adjust p (FDR)	Allele p-value	Allele adjust p	OR (95% CI)	Dominant model adjust p (p-value)	OR (95% CI)	Recessive model adjust p (p-value)	OR (95% CI)
SIRT1	rs12778366 C > T	0.4671	0.519	0.2889	0.3611	0.787 (0.505–1.226)	0.4574 (0.4117)	1.229 (0.751–2.012)	0.728 (0.2715)	0.407 (0.078–2.127)
	rs3758391 T > C	0.9332	0.09332	0.8402	0.8402	0.967 (0.695–1.344)	0.756 (0.756)	0.935 (0.614–1.425)	0.933 (0.9227)	0.963 (0.450–2.061)
	rs3740051 A > G	0.0674	0.2269	**0.0212**	**0.0832**	0.445 (0.220–0.901)	**0.0809** (0.0263)	0.445 (0.215–0.922)	0.728 (0.3083)	3.123 (0.126–77.196)
	rs3818292 A > G	**0.0341**	0.2269	**0.009783**	**0.0832**	0.409 (0.204–0.822)	**0.0809** (0.01176)	0.406 (0.198–0.833)	0.728 (0.3083)	3.123(0.126–77.196)
SIRT2	rs10410544 C > T	0.2355	0.2944	0.3382	0.3758	0.865 (0.642–1.165)	0.1466 (0.1173)	0.689 (0.432–1.099)	0.9329 (0.9329)	0.979 (0.591–1.620)
SIRT6	rs350843 A > C	0.1206	0.2269	**0.0453**	**0.0880**	1.613 (1.007–2.643)	**0.0809** (0.04056)	1.719 (1.020–2.897)	0.728 (0.5824)	0.515 (0.046–5.729)
	rs350844 A > G	0.132	0.2269	0.05113	**0.0880**	1.593 (0.995–2.552)	**0.0809** (0.04534)	1.680 (1.008–2.801)	0.728 (0.5824)	0.515 (0.046–5.729)
	rs107251 T > C	0.1588	0.2269	0.06163	**0.0880**	1.574 (0.975–2.539)	**0.0809** (0.05662)	1.650 (0.983–2.770)	0.728 (0.5824)	0.515 (0.046–5.729)
	rs350845 A > G	0.1588	0.2269	0.06163	**0.0880**	1.574 (0.975–2.539)	**0.0809** (0.05662)	1.650 (0.983–2.770	0.728 (0.5824)	0.515 (0.046–5.729)
	rs350846 C > G	0.06889	0.2269	**0.02496**	**0.0832**	1.734 (1.067–2.818)	**0.0809** (0.02096)	1.846 (1.093–3.120)	0.728 (0.5824)	0.515 (0.046–5.729)

*OR* odds ratio, *CI* confidence interval.

Marginal associations with PD risk are indicated in bold.

**Figure 1:**
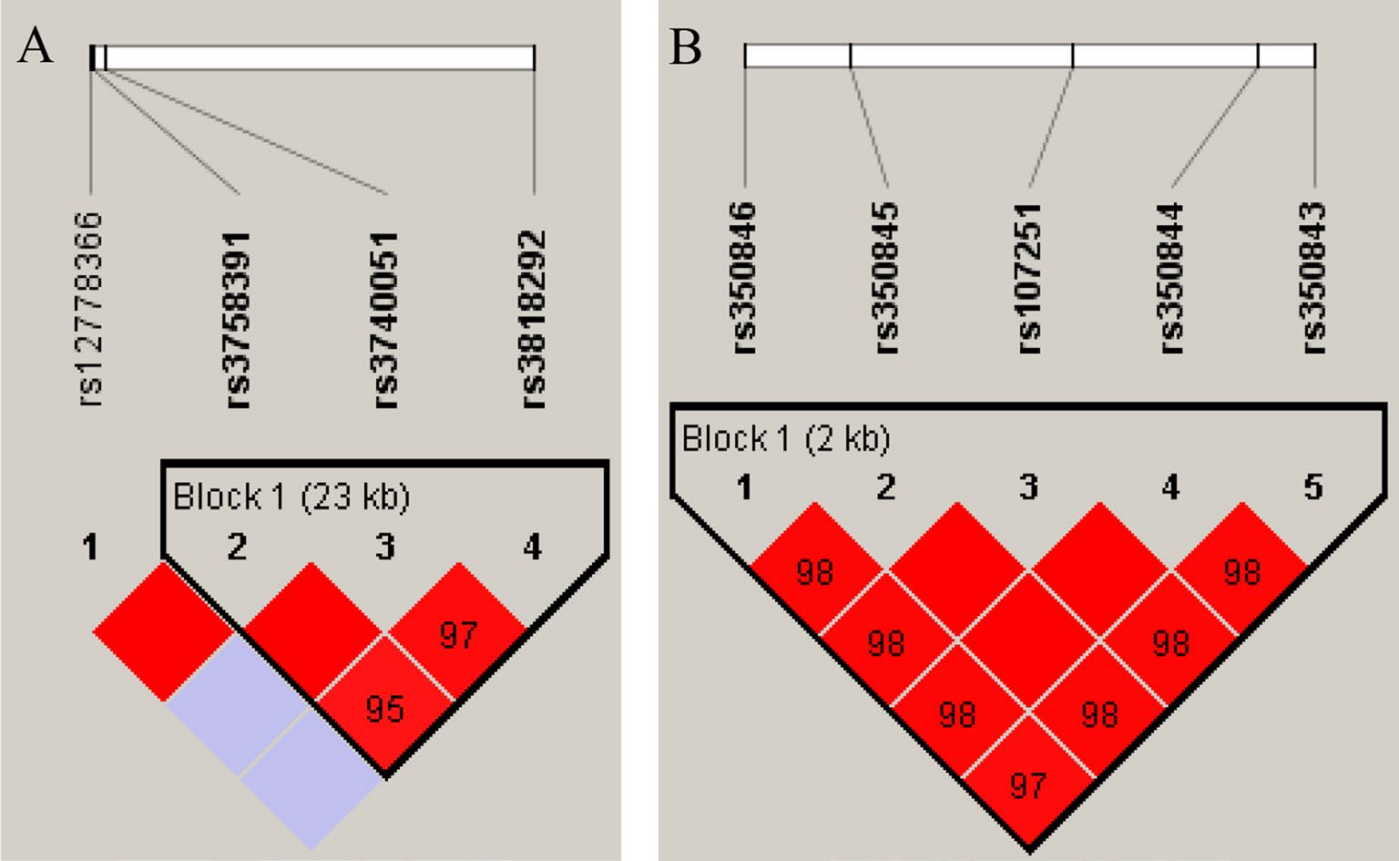
Linkage disequilibrium (LD) plots of single nucleotide polymorphisms (SNPs) of SIRT1 (**A**) and SIR6 (**B**) genes. Shading of diamonds and numbers represent LD between markers based on the D’ values.

Haplotype analysis of the investigated SNPs revealed two haplotype blocks, one in both *SIRT1* and *SIRT6* genes. Strong linkage disequilibrium (LD) was detected between rs3758391, rs3740051 and rs3818292 in *SIRT1* and rs350843, rs350844, rs107251, rs350845 and rs350846 in the *SIRT6* gene (Fig. 1). In the haplotype-based case–control analysis, the TGG haplotype in *SIRT1*and the AATAC haplotype in *SIRT6* showed a trend of association with PD (p = 0.0767 in both cases). The frequencies of other haplotypes showed no significant differences between the two groups (Table 2). Comparison of subgroups (stratified by age at disease onset or gender) did not reveal significant differences in the haplotype frequencies.

**Table 2:** Haplotype frequencies in PD cases and controls.

Haplotype	PD case (freq)	Control (freq)	Chi	p-value	Adjust p
**SIRT1**					
CAA	0.720	0.709	0.107	0.744	0.743
TAA	0.246	0.209	1.339	0.247	0.309
TGG	0.034	0.070	4.667	**0.0307**	**0.0767**
**SIRT6**					
CGCGG	0.856	0.903	3.709	0.0541	0.090
AATAC	0.136	0.082	5.161	**0.0231**	**0.0767**

Haplotype block 1 is identified by rs3758391–rs3740051–rs3818292 in *SIRT1* gene, and haplotype block 2 is identified by rs350843–rs350844–rs107251–rs350845–rs350846 in *SIRT6* gene.

### Associations between PD and SIRT mRNA levels

**Figure 2:**
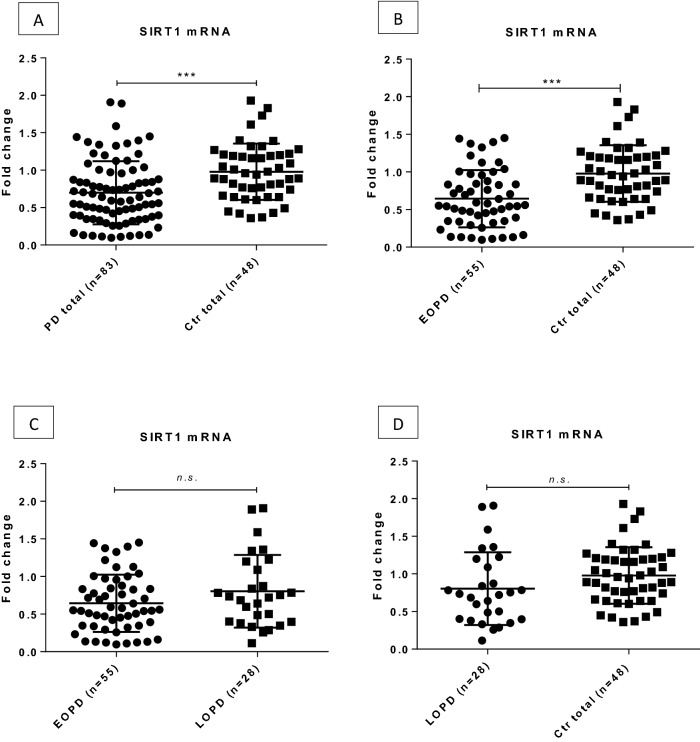
Expression level of SIRT1 in the peripheral blood of PD patients and healthy controls. Significant down-regulation of SIRT1 expression was measured in PD group compared to controls (**A**), and in EOPD patients compared to controls (**B**) as well. No significant difference was detected in the case of EOPD *vs.* LOPD (**C**) and LOPD *vs*. control group (**D**) comparisons. Fold changes are shown with standard deviation. *PD* Parkinson’s disease, *Ctrl* control, *EOPD* early onset Parkinson’s disease, *LOPD* late onset Parkinson’s disease, *n.s.* non-significant; ***p < 0.001 after FDR correction.

In order to determine whether differences in the expression of SIRT genes are detectable when comparing PD and control samples, and whether the presence of any of the studied SNPs has an effect on the expression of the corresponding SIRT gene, we compared the levels of *SIRT1*, *SIRT2* and *SIRT6* mRNAs in peripheral blood samples of PD patients and healthy controls. We found that the level of *SIRT1 * mRNA was significantly reduced in PD patients compared to controls (fold change = 0.71, p = 0.0002). Moreover, *SIRT1* expression was significantly down-regulated in the EOPD cohort as compared to healthy controls (fold change = 0.66, p = 0.00018). There was no significant difference between EOPD and LOPD (fold change = 0.88 p = 0.105) or control and LOPD (fold change = 0.82 p = 0.105, Fig. 2) groups in the levels of *SIRT1* mRNA.

**Figure 3:**
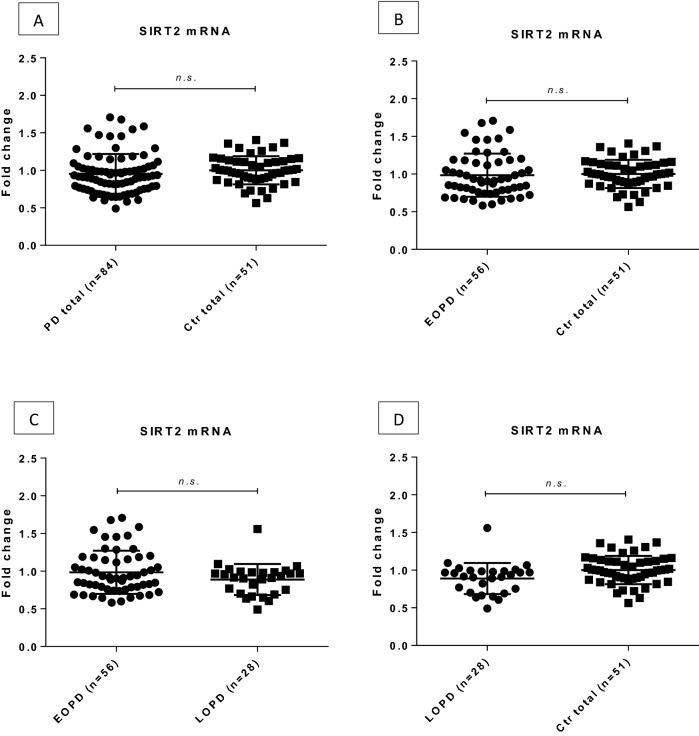
SIRT6 expression levels in peripheral blood of PD patients and controls. SIRT6 expression was significantly increased in PD patients compared to controls (**A**). The up-regulation was more pronounced in EOPD group compared to controls (**B**). No significant difference was detected between EOPD *vs.* LOPD (**C**) and LOPD *vs*. control group (**D**). Fold changes are shown with standard deviation. *PD* Parkinson’s disease, *Ctrl* control, *EOPD* early onset Parkinson’s disease, *LOPD* late onset Parkinson’s disease, *n.s.* non-significant; **p < 0.01 after FDR correction.

On the contrary, *SIRT6* mRNA levels of PD cases were significantly higher compared to healthy controls (fold change = 1.14, p = 0.0078). Furthermore, *SIRT6* was significantly up-regulated in the EOPD group compared to controls (fold change = 1.17, p = 0.0078, Fig. 3). Comparison of EOPD and LOPD subgroups or control group and LOPD patients (fold change = 0.98, p = 0.554 and fold change = 1.1, p = 0.086, respectively) yielded no significant difference (Fig. 3).

**Figure 4:**
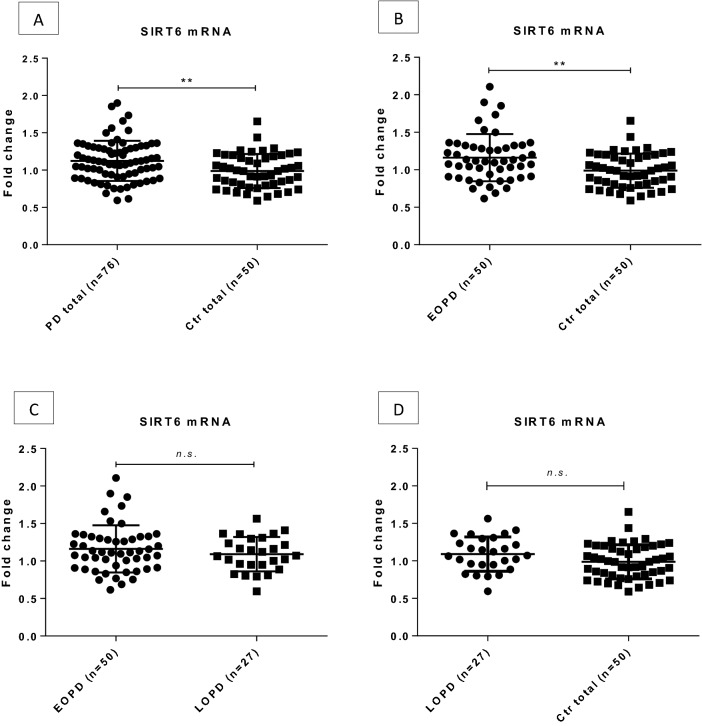
Expression levels of SIRT2 in the peripheral blood of PD patients and healthy controls. The expression level did not shown differences between the investigated groups. Fold changes are shown with standard deviation. *PD* Parkinson’s disease, *Ctrl* control, *EOPD* early onset Parkinson’s disease, *LOPD* late onset Parkinson’s disease, *n.s.* non-significant.

Similarly, no significant difference was observed in *SIRT2* mRNA levels between patient and control groups (fold change = 0.95, p = 0.102, Fig. 4).

Analysis of mRNA levels in relation to the presence of different alleles did not reveal any association either in the PD or in the control group.

## Discussion

There is an increasing body of evidence showing that sirtuins are essential factors in delaying cellular senescence and extending lifespan by the regulation of various cellular processes. It was reported that overexpression of some sirtuins suppress cellular senescence via delaying the age-related telomere shortening and promoting DNA damage repair mechanisms. These data suggest that due to their involvement in various signalling pathways sirtuins are critical modulators of aging thus play an important role in age-related diseases such as neurodegenerative disorders^22^.

In recent years a growing body of evidence has accumulated on the importance of SIRTs in PD pathogenesis^6^. SIRT1 and SIRT2 seem to have opposite effects on the development and course of the disease^21^. A recent meta-analysis revealed strong association between *SIRT6* polymorphisms and PD, suggesting a pathogenic role of SIRT6 in the disease^17^. However, apart from these reports, data regarding the role of the genetic variants of SIRTs in Caucasian populations are scarce.

By the present study, we aimed to assess the frequency of a total of 10 SNPs of *SIRT1, -2* and *-6* genes in Hungarian SPD patients and healthy controls. Furthermore, we aimed at determining if changes in the expression levels of *SIRT1, -2* and *-6* genes are observable between PD patients and controls and also in relation to the presence of the studied SIRT gene variants. All investigated SNPs are located in putative functional regions of the genes and/or have been reported to be associated with several diseases.

SIRT1 regulates numerous physiological processes and its involvement in the pathogenesis of various diseases is well grounded^23^.

In the present work we identified a marginal association between the presence of two *SIRT1* SNPs (rs3740051 and rs3818292) and PD risk. The frequencies of minor (G) allele of both SNPs were higher in the control group, suggesting a protective effect against the development of the disease. In addition, we detected significant down-regulation in *SIRT1* mRNA level in blood samples of PD patients compared to controls. The difference was most prominent when comparing the EOPD group to the control cohort. It is well known that there are some genetic markers which consistently show association with age of onset in PD. Several mutations in parkin, *PINK-1*, leucine-rich repeat kinase 2 and glucocerebrosidase genes are associated with EOPD^24–29^. These observations suggest that the underlying molecular mechanisms of EOPD differ from that of LOPD. However, it seems that the mutations of these genes alone may not be enough to differentiate EOPD from LOPD due to their differing prevalence in different populations. Our results suggest that changes in the expression of *SIRT1* affect PD development by influencing the time of disease onset. Thus variations in the level of *SIRT1* mRNA in the periphery might serve as a potential biomarker for EOPD.

Recently, Chang et al. reported an association between the occurrence of PD and the rs12778366 SNP in Han Chinese population. Chang and colleagues proposed that rs12778366, which is localized in the promoter region of *SIRT1,* might influence the expression of the gene^21^. Our results show no significant association between the rs12778366 SNP and PD, and we detected no effect of the minor allele on gene expression. This could partly be explained by the different allele distribution and contribution to the disease in different study populations.

SIRT2 is a key modulator of many cellular processes such as cell cycle, myelinisation, antioxidant mechanisms and aging^30^. Therefore the role of SIRT2 in neurodegenerative diseases is an intensively studied area of research attracting increasing interest^31–35^. Previous studies indicated that the presence of the T allele of the rs10410544 *SIRT2* variant increased the risk of AD development in both Chinese and Caucasian populations^36,37^. In 2017, Singh et al. reported that over-expression of *SIRT2* in an in vitro model of the disease reduced the formation of alpha-synuclein aggregates. Furthermore, elevated SIRT2 protein activity was measured in the brain of AD and PD patients, suggesting a possible compensatory mechanism against neuronal stress and cell death^15^. More recently elevated serum SIRT2 level was measured in PD patients relative to controls. The increase of SIRT2 level was found to significantly correlate with UPDRS and disease duration among patients with early-phase PD^38^. In this study we investigated whether the AD susceptibility factor rs10410544 polymorphism exerts an effect on *SIRT2* mRNA level in PD patients. Our results did not show any association between the presence of this *SIRT2* SNP and PD, and there was no significant difference detectable in the mRNA levels of patients’ and control groups. These data are in accord with results obtained from Spanish and Chinese populations, which showed no association between the presence of the minor allele of the rs10410544 SNP and PD^20,21^. However, the expression level of *SIRT2* was not investigated in the studies mentioned above. Recently a significant association was reported between the *SIRT2* allele containing rs2015 polymorphism and PD risk in Chinese population. *SIRT2* expression was found to be significantly increased among patients with TT genotype compared to those homozygous for the G allele, suggesting a disease modifying effect of the polymorphism^21^. Based on previous findings of SIRT2 over-expression inducing dopaminergic cell death while enzyme inhibition exerting neuroprotective effects, it was concluded that elevated SIRT2 levels contribute to higher PD risk. In contrast to previous studies reporting on SIRT2 effects on microtubule stability, formation of alpha-synuclein aggregation, neuroinflammation and autophagy^39^ our results do not support the role of SIRT2 in PD.

SIRT6, similarly to other sirtuins, regulates different molecular functions related to DNA repair, tumorgenesis, neurodegeneration and aging^40^. Recently Nicholatos et al. found that SIRT6 knock out cells were more resistant to apoptosis^17^. This study also reported a significant increase in *SIRT6* expression in PD patients’ brain. The meta-analysis of two GWA studies revealed six *SIRT6* SNPs to be associated with increased *SIRT6* mRNA levels and also with increased risk of PD^17^. In line with previous findings of increased SIRT6 expression in *post mortem* PD brain samples, we detected elevated *SIRT6* level in peripheral blood leukocytes of PD patients as compared to controls. Moreover, we found that 5 SNPs, which form a LD block in the N-terminus of the *SIRT6* gene, have a marginal PD risk increasing effect. The association between allele frequencies, mRNA level and PD was more pronounced in the EOPD group. These data emphasize the significance of SIRT6 in the disease, particularly in EOPD cases.

Though an intergroup difference in SIRT6 mRNA level was detected, the low number of samples belonging to particular genotypes prevented us from establishing associations between the different genotypes and mRNA levels. To our knowledge, this is the first report which compared the level of *SIRT6* mRNA in easily accessible peripheral blood cells of PD patients and healthy controls. Elevation in *SIRT6* expression in brain tissue of PD patients has been reported recently^17^. Our finding that the change in *SIRT6* level is detectable also in peripheral blood samples might open possibilities to find new potential markers of diagnostic tests for PD.

In conclusion, we analysed the presence of 10 SNPs of *SIRT1, 2* and *6* genes in Hungarian PD patients for the first time. We observed association of two *SIRT1* SNPs (rs3740051 and rs3818292) and five *SIRT6* variants (rs350843, rs350844, rs107251, rs350845 and rs350846) with PD risk. Additionally, we detected down-regulated *SIRT1* and up-regulated *SIRT6* mRNA levels in peripheral blood of PD patients. Our results, in line with data of others’, strenghten the involvement of SIRTs in the pathogenesis of PD.

Whether the presence of the variants of these genes and their expression changes are in causal relationship with the disease, need further elucidation. Research focusing on this matter is highly warranted: considering the cellular functions SIRT1 and SIRT6 fulfill, gaining insight into their role in the disease can lead to a better understanding of the underlying pathomechanisms of PD and also help in the identification of new therapeutic targets.

## Material and methods

### Subjects

Frequencies of SIRT gene variants were investigated in a cohort consisting of 177 SPD patients (age 64.8 ± 9.9 years, male:female ratio 87:90) and a group of 171 healthy controls (age 62.9 ± 10.9 years, male:female ratio 76:95). For data analysis, the patient’s group was divided into two subgroups based on the appearance of the first symptoms: the early-onset (EOPD; disease onset ≤ 60 years) group comprised of 104, the late-onset (LOPD; disease onset > 60 years) group composed of 73 patients (Supplementary Table S1).

Samples of 84 PD patients (age 62.3 ± 9.8 years) and 52 healthy controls (age 60.3 ± 14.4 years) were involved from the above described groups for comparison of mRNA levels (Supplementary Table S2).

PD was diagnosed based on medical history and physical examination by movement disorder specialists. All those involved in the study were patients under the care of the Department of Neurology, University of Szeged. Patients with secondary parkinsonism were excluded. Individuals of the control group had no history of neurological or psychiatric disorders. All study participants were of Hungarian origin. The study protocol was approved by the Medical Research Council Scientific and Research Ethics Committee (47066-3/2013/EKU (556/2013)) and Ethics Committee of the Faculty of Medicine (22/2012), University of Szeged. All study participants gave written informed consent in accordance with the Helsinki Declaration.

### Genotyping

Genomic DNA was extracted by a standard desalting method^41^ from peripheral venous blood. The extracted DNA was stored at − 20 °C until further use. The analysis of SIRT gene variants was performed with TaqMan allelic discrimination assays obtained from Thermo Fisher Scientific (Thermo Scientific, Waltham, MA, USA).

### RNA extraction and analysis

For RNA analysis 5 ml of venous blood was collected between 8 a.m. and 11 a.m. Samples were treated with Trizol reagent (MRC Inc., Cincinnati, OH, USA) within 2 h of collection, and were kept on − 80 °C until further processing. Total RNA was isolated according to the manufacturer’s protocol (MRC Inc., Cincinnati, OH, USA). RNA concentrations were determined using MaestroNano spectrophotometer (MaestroGenInc, Hsinchu, Taiwan). cDNA was synthesized from 1 μg total RNA with random hexamer primers using RevertAid First Strand cDNA Synthesis Kit (Thermo Scientific, Waltham, MA, USA). The mRNA levels of *SIRT1, SIRT2* and *SIRT6* were determined by RT-PCR using SYBR Green Mastermix (PCR Biosystem Ltd., London, UK). *GAPDH* served as an internal reference gene. The primer efficiencies are plotted in Supplementary Fig. S1. RT-PCR was performed on CFX96 Real Time System (Bio-Rad Laboratories, Hercules, CA, USA).

### Statistical analysis

All study groups involved in the analysis of SNP frequencies were tested for HWE. Chi-square or Fisher’s exact test was used to compare the genotype frequencies and allele distributions between cases and controls. Associations between genotypes and PD were estimated via odds ratio (OR) with 95% confidence interval (CI). Genotype frequencies were compared between cases and controls under the additive, dominant and recessive model.

The relative mRNA level was calculated by the 2^−ΔΔCt^ method^42^. For the identification of the outliers among 2^−ΔΔCt^ replicates the ROUT method was implemented. D’Agostino and Pearson omnibus normality test was performed for the analysis of data distribution. If the data showed Gaussian distribution, we implement unpaired t-test. In the case of non-Gaussian distribution Mann–Whitney U test was used for the comparison of the relative levels of mRNAs between different groups. Due to multiple comparisons FDR correction was implemented. A p value less than 0.05 was considered statistically significant. Statistical analyses were performed using PLINK, R and GraphPad Prism 6.0 softwares. LD and haplotype-based case–control analysis were performed with the use of Haploview 4.2 software.

### Ethics approval and consent to participate

The study protocol was approved by the Medical Research Council Scientific and Research Ethics Committee (47066-3/2013/EKU (556/2013)) and Ethics Committee of the Faculty of Medicine (22/2012), University of Szeged. All study participants gave written informed consent in accordance with the Helsinki Declaration.

## Supplementary Information

Supplementary Information.

## Data Availability

The data used in the current study are available from the corresponding author on reasonable request.
