# Supplementary material for: Gene variants and expression changes of SIRT1 and SIRT6 in peripheral blood are associated with Parkinson’s disease
Source: Sci Rep. 2021 May 21;11:10677. doi: 10.1038/s41598-021-90059-z (PMC8140123; doi:10.1038/s41598-021-90059-z)
Supplement: Supplementary file 1 — Supplementary Information. [file 41598_2021_90059_MOESM1_ESM.docx]

**Supplementary file**

Gene variants and expression changes of SIRT1 and SIRT6 in peripheral blood are associated with Parkinson’s disease

Rita Maszlag-Török^a^, Fanni A. Boros^a^, LászlóVécsei^a,b^, PéterKlivényi^a,*^

^a^Department of Neurology, Albert Szent-Györgyi Clinical Center, Faculty of Medicine, University of Szeged, Szeged, Hungary;

^b^MTA - SZTE Neuroscience Research Group, Szeged, Hungary

^*^Corresponding author:

Péter Klivényi, MD, PhD, DSc

Department of Neurology, Albert Szent-Györgyi Medical Center, Faculty of Medicine, University of Szeged

P.O. Box: 427, H-670l, Szeged, Hungary

Tel/Fax: +36 62545-351, +36 62545-597;

E-mail: [klivenyi.peter@med.u-szeged.hu](mailto:klivenyi.peter@med.u-szeged.hu)

|  | PD case | Control |
| --- | --- | --- |
| No. | 177 | 171 |
| Age (mean±SD; years) | 64.8 ±9.9 | 62.9 ± 10.9 |
| Male/female ratio | 87/90 | 76/95 |
| Disease onset  EOPD No.  Age(mean±SD; years)  Disease duration(mean±SD; years)  LOPD No.  Age(mean±SD; years)  Disease duration(mean±SD; years) | 104  59.3±8.8  8.9±6.2  73  72.5±4.9  4.9±4.1 |  |

Supplementary table 1. Demographic data of the SNP study groups. Abbreviations: PD: Parkinson's disease; EOPD: early-onset Parkinson's disease; LOPD: late-onset Parkinson's disease; SD: standard deviation.

|  | PD case | Control |
| --- | --- | --- |
| No. | 84 | 52 |
| Age (mean±SD; years) | 62.3±9.8 | 60.3±14.4 |
| Male/female ratio | 44/40 | 21/31 |
| Disease onset  EOPD No.  Age(mean±SD; years)  Disease duration(mean±SD; years)  LOPD No.  Age(mean±SD; years)  Disease duration(mean±SD; years) | 56  57.9±8.4  7.8±4.8  28  71.2±5.3  5.7±4.2 |  |

Supplementary table 2. Demographic data of the PD patients and healthy controls involved in gene expression analysis. Abbreviations: PD: Parkinson's disease; EOPD: early-onset Parkinson's disease; LOPD: late-onset Parkinson's disease; SD: standard deviation.


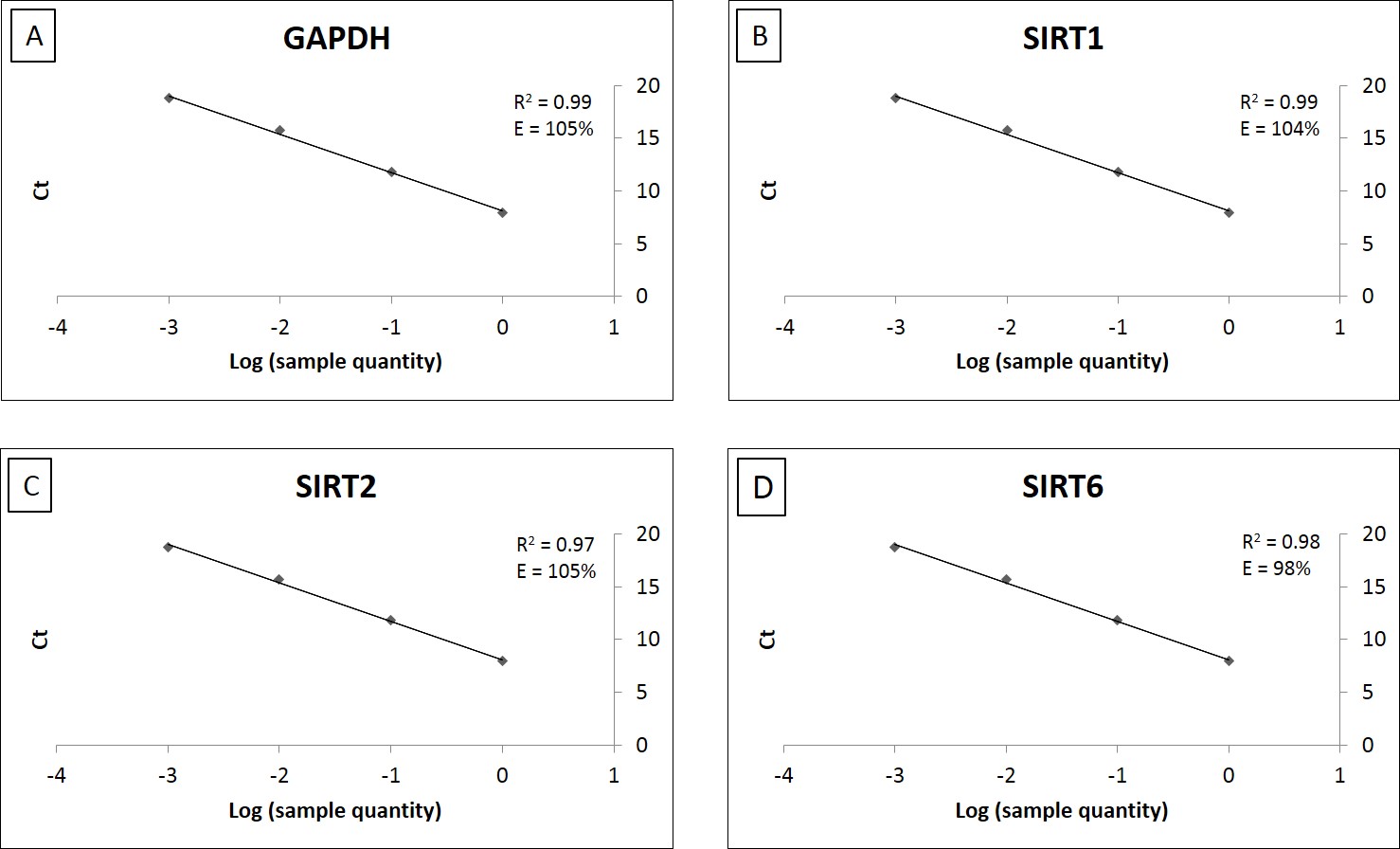


Supplementary figure 1. Evaluation of RT-qPCR primer efficiencies for the four genes indicated on the plots. Four steps of 10-fold serial dilutions of cDNA samples were used to determine primer efficiencies by standard curve generation. PCR reactions with each primer pairs were run separately and Ct values versus dilution factor are plotted in a base-10 semi-logarithmic graph. Correlation coefficients (R^2^) and calculated efficiencies (E) are indicated. In our study for RT-qPCR analysis 10-fold diluted cDNA samples were used (corresponding to -1 log sample quantity on the graphs).
